# *Phaeocystis globosa Virus* DNA Polymerase X: a “Swiss Army knife”, Multifunctional DNA polymerase-lyase-ligase for Base Excision Repair

**DOI:** 10.1038/s41598-017-07378-3

**Published:** 2017-07-31

**Authors:** José L. Fernández-García, Ana de Ory, Corina P. D. Brussaard, Miguel de Vega

**Affiliations:** 1 Centro de Biología Molecular Severo Ochoa (CSIC-UAM), C/Nicolás Cabrera 1, Campus Cantoblanco, 28049 Madrid, Spain; 2 Department of Marine Microbiology and Biogeochemistry, NIOZ Royal Netherlands Institute for Sea Research, Utrecht University, NL-1790 AB Den Burg (Texel), The Netherlands

## Abstract

*Phaeocystis globosa* virus 16T is a giant virus that belongs to the so-called nucleo-cytoplasmic large DNA virus (NCLDV) group. Its linear dsDNA genome contains an almost full complement of genes required to participate in viral base excision repair (BER). Among them is a gene coding for a bimodular protein consisting of an N-terminal Polβ-like core fused to a C-terminal domain (PgVPolX), which shows homology with NAD^+^-dependent DNA ligases. Analysis of the biochemical features of the purified enzyme revealed that PgVPolX is a multifunctional protein that could act as a “Swiss army knife” enzyme during BER since it is endowed with: 1) a template-directed DNA polymerization activity, preferentially acting on DNA structures containing gaps; 2) 5′-deoxyribose-5-phosphate (dRP) and abasic (AP) site lyase activities; and 3) an NAD^+^-dependent DNA ligase activity. We show how the three activities act in concert to efficiently repair BER intermediates, leading us to suggest that PgVPolX may constitute, together with the viral AP-endonuclease, a BER pathway. This is the first time that this type of protein fusion has been demonstrated to be functional.

## Introduction

DNA base lesions are the most common type of genomic damage and pose a challenge to genome stability. Lesions can arise after exposure of DNA to genotoxicants [e.g., radiation, alkylating mutagens, by-products of cellular metabolism such as reactive oxygen species (ROS)], or can arise spontaneously under physiological conditions, such as the hydrolysis of the glycosidic bonds that leaves uninformative apurinic/apyrimidinic (AP) sites, and deaminations that lead to miscoding bases^1, 2^. The base excision repair (BER) pathway is a multi-step enzymatic pathway that is primarily responsible for repairing a broad spectrum of non-bulky and non-helix-distorting DNA lesions produced by the oxidation, alkylation, deamination or hydroxylation of DNA bases^3^. The general pathway consists of five steps: 1) recognition and hydrolytic cleavage of altered base-sugar bonds by DNA *N*-glycosylases^4^; 2) recognition of the resulting AP sites by an AP-endonuclease, which cleaves at the 5′-side of the AP site to render a 1-nt gap flanked by 3′-OH and 5′-dRP termini; 3) removal of the 5′-dRP moiety by a 5′-dRP lyase, leaving a ligatable 5′-P end; 4) short gap filling by a specific DNA polymerase to restore the original (nondamaged) nucleotide; and 5) final nick sealing by a DNA ligase (reviewed in ref. 3).

Family X DNA polymerases (PolXs), including mammalian Pol β^5^ and Pol λ^6, 7^, bacterial PolXs^8–10^ as well as the African Swine Fever Virus (ASFV) PolX^11, 12^, are involved in the filling-in step of BER. These polymerases are relatively small, monomeric proteins that catalyze the insertion of a few nucleotides and lack an intrinsic proofreading activity^13^. Their optimized architecture allows them to efficiently accomplish the filling-in of short gaps that arise in BER. In general, PolX members present a common Polβ-like core^14^ with an N-terminal 8-kDa domain that specifically recognizes the 5′-phosphate group of the gap, which allows the correct positioning of the enzyme on the gapped or nicked structure^15–17^. The 8-kDa domain of several eukaryotic PolXs including Pol β and Pol λ^18, 19^ and yeast Pol4^20^ and Trf4^21^, is also endowed with a 5′-dRP lyase activity that removes the 5′-dRP group during short-patch BER^6, 22^. The C-terminal polymerization domain of the Polβ-like core possesses the three universally conserved subdomains, fingers, palm and thumb, which are responsible for the binding and further elongation of the 3′ terminus of the upstream primer strand^14^. The only known exception to this structural generality is the ASFV PolX, which lacks the 8-kDa domain and the fingers subdomain^11^.

In several PolXs, the domain responsible for the polymerization reaction is fused to catalytic and/or protein-protein interaction domains. Thus, the Polβ-like core of the eukaryotic DNA polymerases λ, μ, terminal deoxynucleotidyl transferase (TdT) and yeast Pol IV, is fused to an N-terminal BRAC1 carboxy terminus (BRCT) domain that interacts with nonhomologous end joining-DNA bound factors to recruit these polymerases to double-strand breaks^23–28^. In Bacterial and Archaeal PolXs, the C-terminus of the Polβ-like core is fused to a polymerase and histidinol phosphatase (PHP) domain^29^, which contains 3′-5′ exonuclease, 3′-phosphatase and 3′-phosphodiesterase activities, and endows the polymerase with the capacity to process damaged 3′ termini^10, 30–32^. It also has an AP-endonuclease activity, which enables the enzyme to process an AP site and restore the original nucleotide^9, 10^.

Aside from bacteria, archaea and eukaryotes, some viruses contain PolXs. The nucleo-cytoplasmic large DNA virus (NCLDV) group constitutes a monophyletic group of viruses that infect a wide range of eukaryotes, such as unicellular marine protists, insects, fish and mammals. They possess dsDNA genomes ranging from 100 kb to 2.5 Mb that, unlike smaller viruses, usually have many genes that encode for putative DNA repair proteins^33, 34^. The genome of the viral strain 16T that infects *Phaeocystis globosa* (strain PgV-16T), a high-biomass-forming phytoplankton species with a central role in oceanic carbon and sulfur cycles^35^, has been recently sequenced^36^. PgV-16T is a giant virus from the *Megaviridae* family^36^ that belongs to the NCLDV group and has a linear dsDNA genome 459,984 bp in length. Its genome contains an almost full complement of genes (Xth/APE1-like AP-endonuclease, a hypothetical PolX and a NAD^+^-dependent DNA ligase) required to execute a potential viral BER pathway^33, 34, 36^. NAD^+^-dependent ligases are present in a minority of NCLDVs and most likely were acquired from a bacteriophage at the early stages of evolution of eukaryotes^33, 37^, whereas PolX could be acquired from different cellular organisms including eukaryotes^33, 38^. Interestingly, whereas PolX and ligase are encoded by two independent genes in the *Marseilleviridae*, *Mimiviridae* and *Poxviridae* families, which also belong to the NCLDV group, PgV-16T and its close relative CeV, a virus infecting the unicellular marine phytoplankton *Haptolina* (formerly *Chrysochromulina*) *ericina*, represent the first examples where the DNA ligase gene is fused to the C-terminus of the PolX coding region^36, 39^.

To expand the current knowledge on the role of PolXs in BER, we have characterized the putative PolX from PgV-16T (PgVPolX). Our results indicate that, in addition to the general polymerization properties shared by most PolXs, PgVPolX has an intrinsic 5′-dRP lyase and NAD^+^-dependent DNA ligase activity. The coordination of these activities enables the enzyme to perform the last three steps of BER: removal of the 5′-dRP moiety, filling-in of the resulting gap, and final sealing of the nick to restore the original genomic information. To the best of our knowledge, this is the first characterization of this type of fused protein.

## Results

### *P. globosa* virus 401 gene codes for a family X DNA polymerase

**Figure 1:**
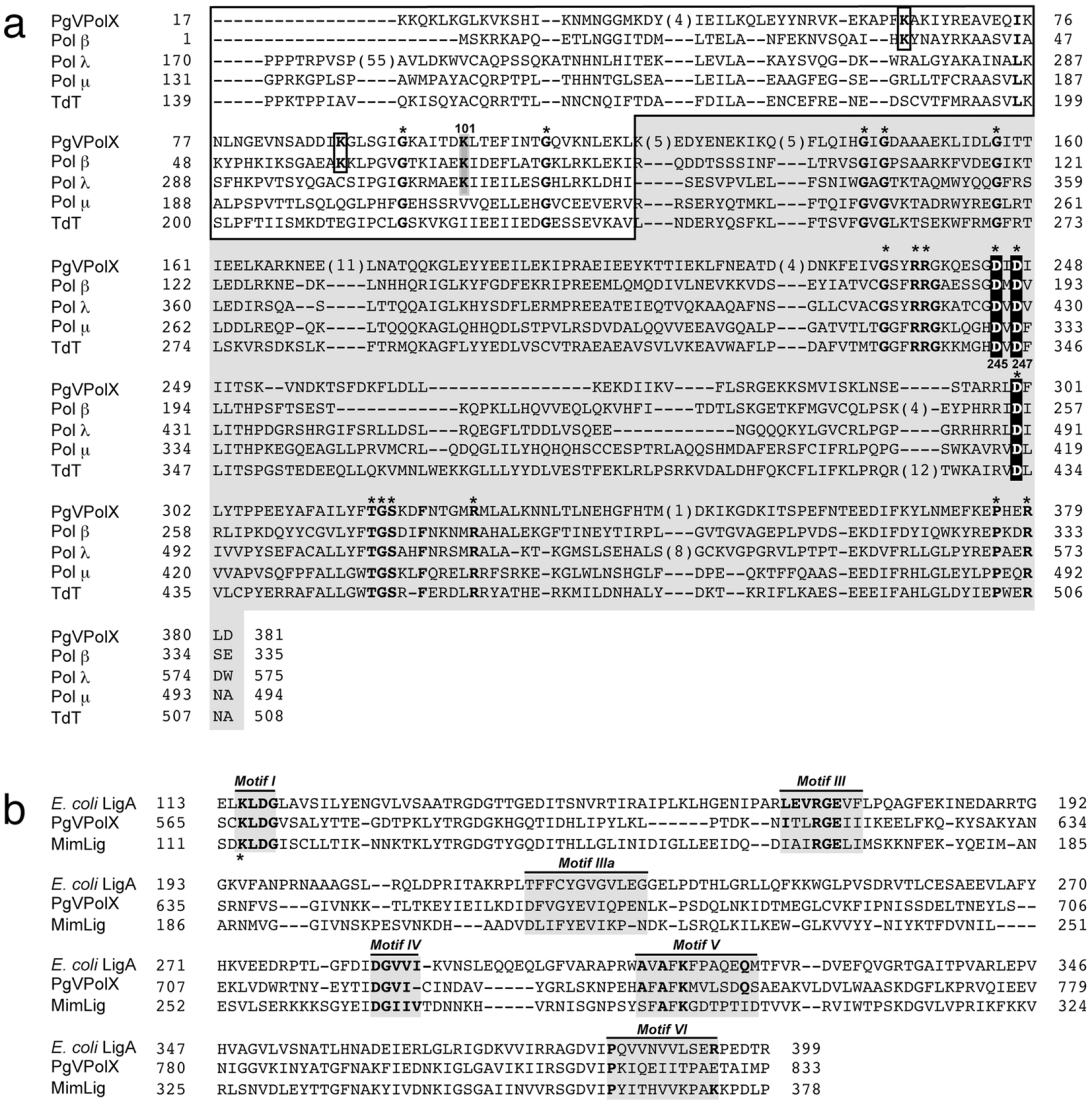
(**a)** Multiple sequence alignment of the Pol β-like core of PgVPolX with human family X DNA polymerases. Numbers indicate the amino acid position relative to the N-terminus of each DNA polymerase. Names of organisms are abbreviated as follows (numbers in parentheses indicate the corresponding accession number): PgV-16T PolX, DNA polymerase X from strain 16 T of *P. globosa* virus (NC_021312.1); Polβ, human DNA polymerase β (NP_002681); Pol λ, human DNA polymerase λ (NP_037406); Polμ, human DNA polymerase μ (NP_001271260.1); TdT, terminal deoxynucleotidyl transferase (NP_001017520.1). White letters boxed in black indicate the three conserved Asp responsible for the polymerization reaction. Black bold letters boxed in gray indicate the nucleophile residue responsible for the 5′-dRP lyase activity in Pol β and Pol λ. Black bold letters boxed in white indicate the other two lysine residues described to play a role in 5′-dRP lyase catalysis in Pol β. Asterisks indicate invariant residues among PolX members^11^. According to Pol β structural data^17, 60^, the alignment is divided into the 8-kDa subdomain (white area), and the polymerization domain (grey area). **(b)** *Alignment of the C-terminal NAD* ^*+*^ *-dependent DNA ligase domain of PgVPolX with the Ligase A from E. coli and Mimivirus DNA Ligase (MimLig)*. The conserved Motifs I-VI present in NAD^+^-dependent DNA ligases are indicated^61^. An asterisk indicates the lysine residue that is specifically adenylated during the ligation reaction. The accession numbers of *E. coli* LigA and MimLig are WP_001413640.1 and Q5UPZ0.1, respectively.

The complete genome sequence of the *P. globosa* virus revealed an ORF corresponding to gene 401, which would encode for a 1093 amino acid protein whose N-terminal 379 amino acids have homology with family X DNA polymerases (30% identity with human Pol β) (Fig. 1a); and whose C-terminal domain (475–1050) has homology with the NAD^+^-dependent DNA ligases^36^ (Fig. 1b). To determine whether this type of fused viral gene is functional, we cloned the entire ORF into the pET16(a)+ expression vector which carries an N-terminal (His)_10_-tag. The recombinant protein was expressed in *Escherichia coli* BL21(DE3) cells and purified as described in Materials and Methods.

**Figure 2:**
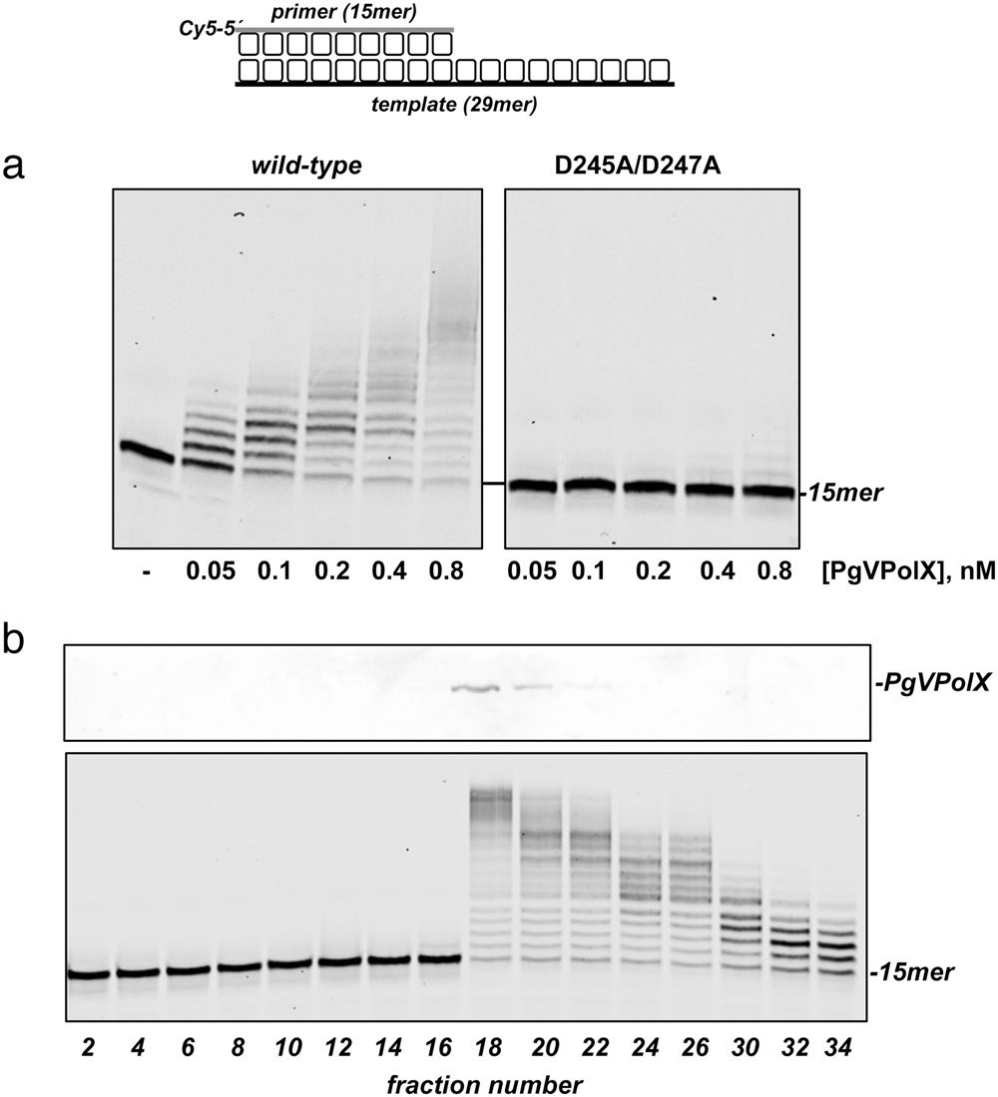
(**a**) Polymerization activity of PgVPolX on a template/primer substrate. The assay was performed as described in Materials and Methods, with 25 nM of the primer/template substrate (schematized on top of the figure) and 100 µM dNTPs. After incubation for 5 min at 30 °C, the reactions were stopped by adding EDTA up to 10 mM. Samples were analyzed by 7 M urea-20% PAGE and visualized using a Typhoon 9410 scanner. The position of the primer is indicated. **(b)** Sedimentation analysis of the polymerization activity of PgVPolX. The upper panel shows the SDS-PAGE analysis followed by Coomasie Blue staining of the even gradient fractions 2–34. The bottom panel shows the polymerization activity of the individual fractions using the primer/template substrate depicted on top of the figure, in the presence of 100 µM dNTPs. Samples were processed as in (**a**).

To examine the presence of a polymerization activity in PgVPolX, the purified protein was incubated with a 5′-Cy5-labeled primer/template DNA (depicted in Fig. 2, *top*), 100 µM dNTPs and Mg^2+^ as a metal activator. As shown in Fig. 2a (*left* panel), PgVPolX catalyzed the 5′-3′ extension of the primer molecule, demonstrating a distributive polymerization activity as it yielded polymerization products whose length was strongly dependent on the enzyme:DNA ratio. This result suggests that PgVPolX is well suited to accomplish short-stretch DNA synthesis *in vivo*. To determine whether the polymerization activity was inherent to PgVPolX, the purified protein was sedimented through a glycerol gradient, and the collected fractions were individually assayed for DNA polymerase activity on the same substrate. A single activity peak cosedimented with the mass peak of the purified PgVPolX (Fig. 2b). Additionally, substitution of the two predicted metal ligands Asp245 and Asp247 with alanine (Fig. 1a) rendered a virtually inactive protein (Fig. 2a, *right* panel), and the residual polymerization activity was intrinsic to the mutant enzyme (Supplementary Fig. S1A). Overall, the results allow us to unambiguously assign a DNA polymerization activity to PgVPolX and rule out the presence of a contaminant DNA polymerase from *E. coli*.

**Figure 3:**
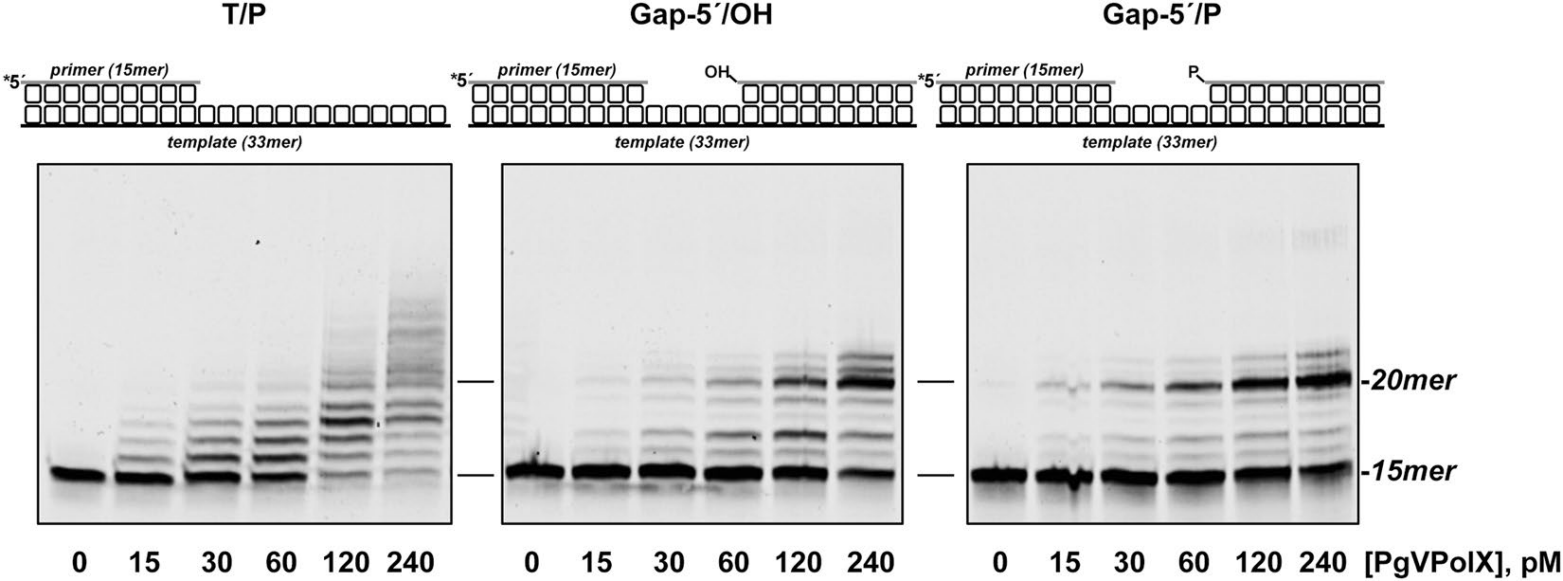
DNA substrate preference for PgVPolX. The following different molecules used in the analysis: *T/P*, template-primer; *Gap-5*′*/OH*, 5 nucleotides gap; and *Gap-5*′*/P*, 5 nucleotides gap bearing a 5′-phosphate group are schematized on top of the figure. The assay was carried out as described in Materials and Methods with 25 nM DNA substrate and 100 µM dNTPs. Asterisks indicate the 5′ Cy5-labeled end of the primer strand.

As expected for a PolX family DNA polymerase, the presence of a downstream oligonucleotide in 5-nt gapped structures promoted the rapid appearance of a band corresponding to the fully repaired gap (Fig. 3, *middle* and *right* panels). The slightly faster repair of the Gap-5′/P substrate (Fig. 3, *right* panel) suggested that recognition of the 5′-P increases the processivity of PgVPolX. In this sense, the fact that most of the T/P substrate was elongated at the highest PolX concentration used (Fig. 3, *left* panel) in comparison with the gapped molecules (Fig. 3, *middle and left* panels) would indicate more frequent dissociation/reassociation events, possibly as a consequence of a less tight binding to the T/P structure. Similar to Pol β, PgVPolX displayed a partial strand displacement capacity and efficiently inserted several additional nucleotides after filling-in the gap.

**Figure 4:**
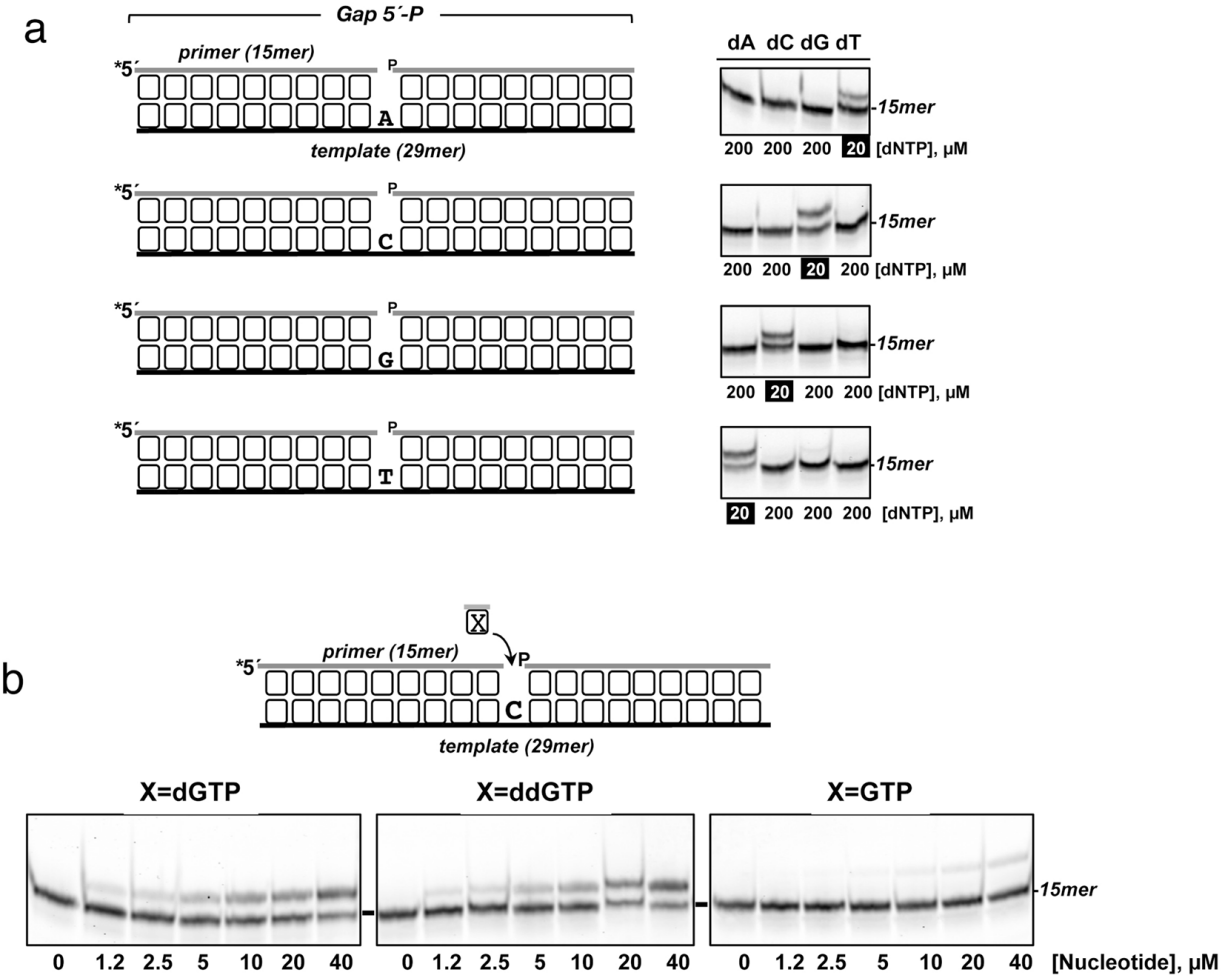
**(a**) *Pg*VPolX is a template-directed DNA polymerase. The four different template-primer structures used, differing in the templating base of the 1-nt gaps, are shown on the left. The assay was performed as described in Materials and Methods with 25 nM of the indicated substrate, 3 nM of PgVPolX and either 20 µM of the correct dNTP or 200 µM of each of the three incorrect dNTPs. **(b)** *Insertion of different types of nucleotides by PgVPolX*. The assay was performed as described in Materials and Methods in the presence of 25 nM of the substrate depicted on the top of the panel, 3 nM of PgVPolX and the indicated concentrations of nucleotides. Asterisks indicate the 5′ Cy5-labeled end of the primer strand.

Having shown that PgVPolX is a DNA-dependent DNA polymerase, we next analyzed its ability to select among the four deoxynucleotides (base discrimination) to catalyze faithful DNA synthesis. Thus, the incorporation of each of the four dNTPs was assayed individually on the four 1-nt gapped DNA structures depicted in Fig. 4a, covering the 16 possible template-substrate nucleotide pairs. As shown in Fig. 4a, PgVPolX initiated DNA synthesis following the Watson-Crick base pairing rules as it extended the primer strand exclusively in the presence of the complementary (correct) nucleotide despite a 10-fold higher concentration of each of the three non-complementary (incorrect) deoxynucleotides. To evaluate 3′- and 2′-OH discrimination by PgVPolX, we used a defined 1-nt gapped DNA molecule to compare dGMP, ddGMP and GMP incorporation. As shown in Fig. 4b, the enzyme inserted dGMP and ddGMP nucleotides with a similar efficiency, suggesting that PgVPolX does not show a strong selection for the 3′-OH group of the nucleotide. Similar to that observed for Pol β and Pol λ, PgVPolX was severely impaired in its ability to incorporate ribonucleotides, most likely due to the presence of the aromatic residue Tyr315 (see Fig. 1a), which is a homolog of residues Tyr271 and Tyr505 of Pol β and Pol λ, respectively, and has been described to discriminate against the ribose 2′-OH group^40, 41^.

### PgVPolX has an inherent NAD^+^-dependent DNA ligase activity

**Figure 5:**
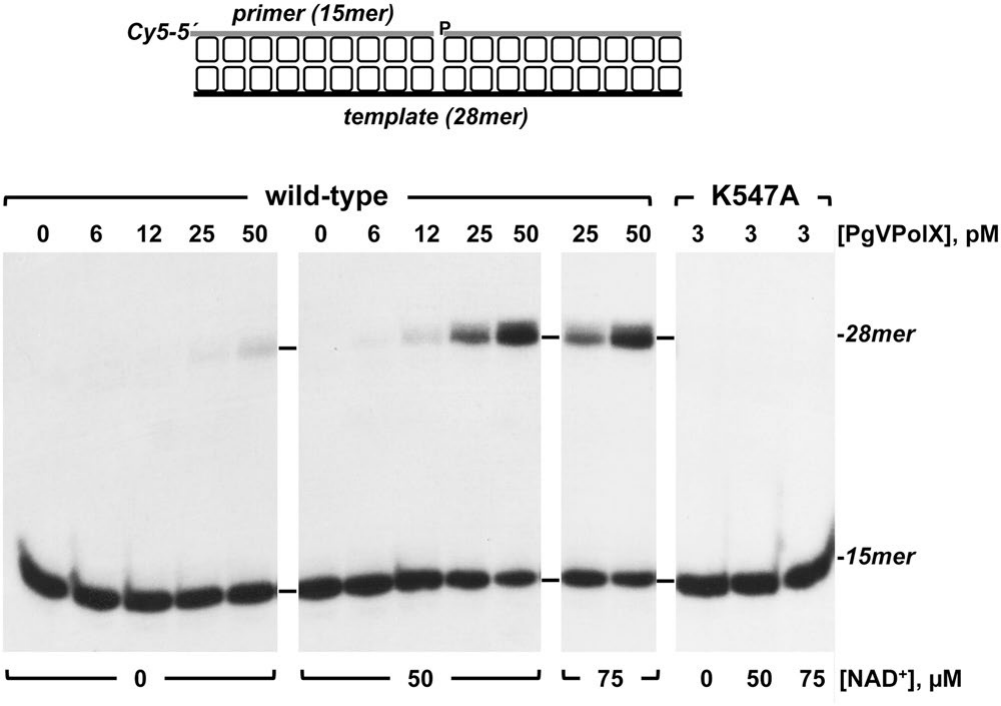
Ligation of nicked DNA by PgVPolX. The assay was performed as described in Materials and Methods with 1 nM of the ^32^P-5′-labeled substrate depicted on top of the figure and the indicated concentrations of either the wild-type or mutant PgVPolX and of NAD^+^.

As mentioned above, the PgVPolX C-terminal residues 475–1050 show homology with NAD^+^-dependent DNA ligases. We therefore assayed the ability of the recombinant PgVPolX to seal a duplex DNA substrate harboring a single nick. As shown in Fig. 5, the enzyme successfully converted the 5′^32^P-labeled 15mer substrate to a 28mer product, confirming the presence of a DNA ligase activity. In addition, the inclusion of 50 µM NAD^+^ in the reaction mixture stimulated nick ligation about 7-fold, in agreement with an NAD^+^-dependent DNA ligase activity. The activity observed in the absence of NAD^+^ is attributed to preadenylated PgVPolX in the enzyme preparation, as described for other DNA ligases^42–44^. As shown in Fig. 1b, the amino acid sequence of PgVPolX contains the KxDG motif I, a signature feature of the ligase superfamily wherein the lysine residue forms a covalent intermediate with AMP, which is further transferred to the 5′-P of the nick before the final sealing step^45^. Thus, to further confirm that the ligase activity observed with PgVPolX was inherent to the purified enzyme, the corresponding Lys547 residue was changed to an alanine (mutant K547A). As expected, the mutation abolished the ligation activity of the protein without affecting its polymerization activity (Supplementary Fig. S1B). This result is consistent with Lys547 as the adenylation site of the protein and eliminates the possibility that a contaminant from the bacterial expression system is responsible for the ligase activity of the wild-type enzyme.

### 5′-dRP Lyase activity associated with PgVPolX

**Figure 6:**
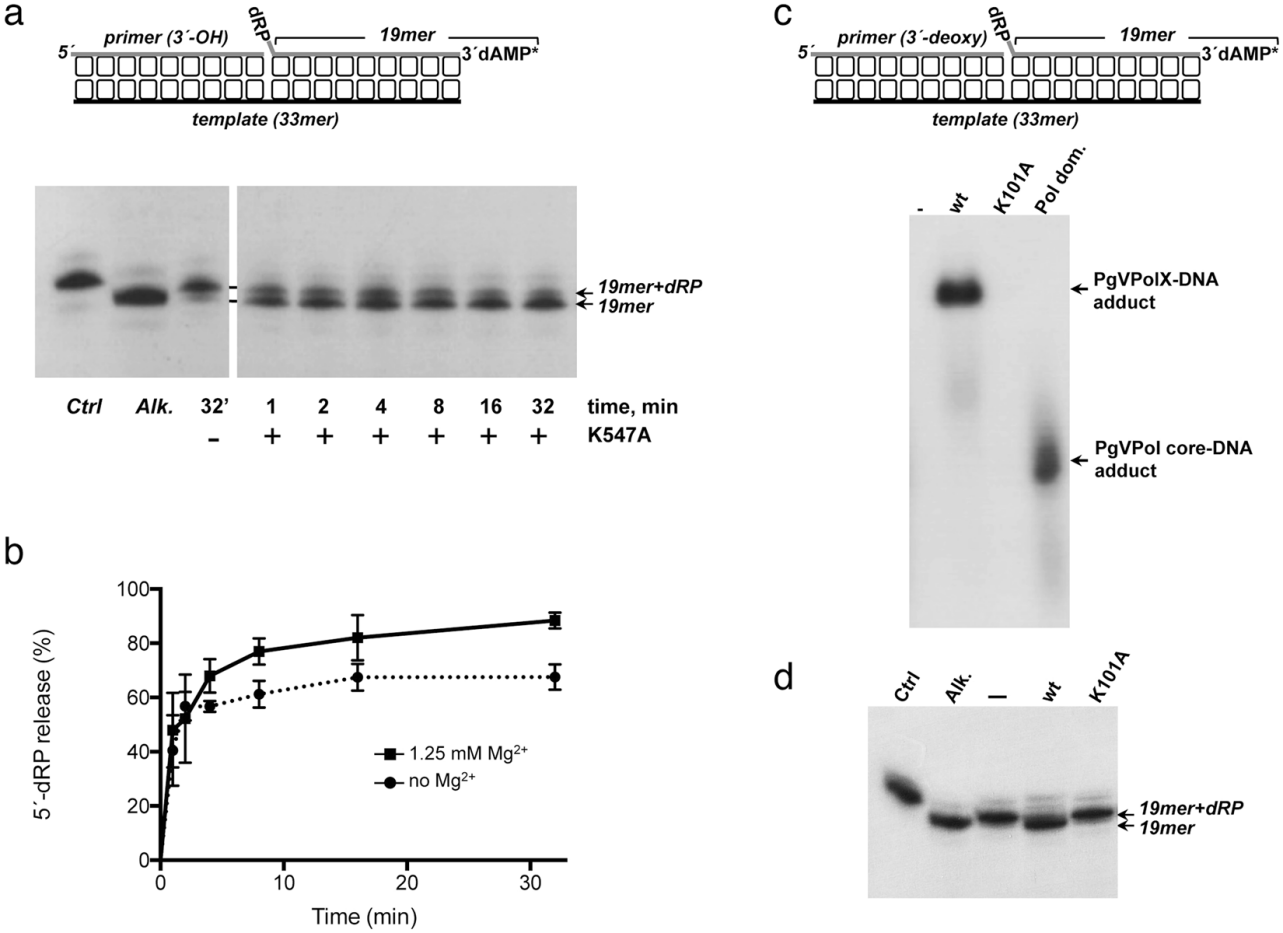
5′-dRP lyase activity of PgVPolX. (**a**) *Time course analysis of the 5*′*-dRP release by PgVPolX*. The assay was performed as described in Materials and Methods with 1 nM of the depicted substrate (top), and 3 nM of the ligase deficient-mutant K547A. After incubation for the indicated times, samples were analyzed by 7 M urea-20% PAGE and further autoradiography. Position of products is indicated. *Alk*. Alkalyne hydrolysis of the 5′-dRP moiety. **(b)** *Quantification of the 5*′*-dRP released by PgVPolX*. The assay was performed as in (a) with 1 nM of the depicted substrate (top), 3 nM of the ligase-deficient mutant K547A in the absence or presence of 1.25 mM MgCl_2_. The values plotted represent the ratio 19mer/(19mer +19mer +dRP) ×100 at each reaction time and are the mean of three independent experiments. (**c**) *Formation of PgVPolX-DNA adducts*. Reactions were performed as described in Materials and Methods, incubating 12 nM of the indicated protein, 6 nM of the depicted [^32^P]3′-labeled DNA substrate (top) and 100 mM NaBH_4_. Samples were analyzed by 10% SDS-PAGE and further autoradiography. (**d**) 5′-dRP lyase activity of PgVPolX relies on residue Lys101. The assay was performed as described in Materials and Methods by incubating 4 nM of the depicted substrate (top of panel c) and 3 nM of the indicated protein. After incubation for 2.5 min, samples were analyzed by 7 M urea-20% PAGE and further autoradiography. *Alk*. Alkalyne hydrolysis of the 5′-dRP moiety.

In addition to single-stranded DNA binding and 5′-phosphate recognition, the 8-kDa domain of Pol β exhibits a 5′-dRP lyase activity responsible for the release of the 5′-dRP moiety during short patch BER^46^. As shown in Fig. 1a, there is significant amino acid similarity between residues 1–116 of PgVPolX and those forming the 8-kDa domain in Pol β (30% identity). More specifically, the residues that play a role in 5′-dRP lyase catalysis in Pol β (Lys35, Lys60, and Lys72^47, 48^) are conserved in PgVPolX (Lys64, Lys89 and Lys101). The Pol β residue Lys72 was identified as the nucleophile responsible for the release of the 5′-dRP group^49^. A homologous lysine residue is present in those PolXs with a 5′-dRP lyase, as in Pol λ (Lys310^18^), but it is absent in those PolXs that lack such an activity, such as Pol μ and TdT (see Fig. 1a). Thus, to evaluate the 5′-dRP release proficiency of PgVPolX, a DNA hybrid mimicking a gap-filled BER intermediate with the upstream 3′-OH end adjacent to the downstream dangling 5′-dRP group was used as a substrate (see Materials and Methods and scheme in top of Fig. 6a), and was incubated with the PgVPolX ligase-deficient mutant K547A to prevent potential further ligations. As shown in Fig. 6a, the PgVPolX K547A mutant removed the 5′-dRP moiety, as detected by the size reduction of the labeled substrate. Under these conditions, the absence of divalent cations did not impede the release of the 5′-dRP group by the protein, pointing to a metal-independent 5′-dRP lyase activity. Although unnecessary, the addition of Mg^2+^ to the reaction improved slightly the release of the 5′-dRP group by PgVPolX (Fig. 6b). We hypothesize that the presence of this metal ion likely assists the stable/proper binding of the protein to the DNA substrate, as described for the 5′-dRP lyase activity of Pol β^19, 48^.

The release of 5′-dRP by DNA polymerases β, λ, γ, ι and θ proceeds through β-elimination, which involves the formation of a Schiff-base intermediate and has allowed categorizing the activity as a 5′-dRP lyase^18, 19, 49–51^. To determine whether this was also the case for PgVPolX, we exploited the ability of NaBH_4_ to reduce a Schiff-base intermediate to form a covalent protein-DNA complex. Accordingly, if the catalytic mechanism of PgVPolX involved a Schiff-base intermediate, addition of NaBH_4_ to a 5′-dRP-containing substrate would permit trapping of a DNA-protein complex that could be detected by autoradiography after separation by SDS-PAGE. As shown in Fig. 6c, PgVPolX formed a stable adduct with a 5′-dRP-containing 19mer strand, indicating that the 5′-dRP removal activity of PgVPolX proceeds through β-elimination. The fact that the purified polymerization domain (residues 1–381) gave rise to an adduct with an electrophoretic mobility faster than that of the complex formed with the complete enzyme indicates that the 5′-dRP lyase activity is intrinsic to PgVPolX and resides in the Polβ-like core of the enzyme.

As mentioned above, Lys72 and Lys310 residues from Pol β and Pol λ, respectively, have been identified as the nucleophiles responsible for the Schiff-base formation during the β-elimination of the 5′-dRP moiety^18, 49^. The amino acid sequence comparison shown in Fig. 1a strongly suggests that PgVPolX residue Lys101 may be the homolog nucleophile in this polymerase. To test this possibility, PgVPolX Lys101 residue was changed into Ala (K101A) and the mutant protein was assayed for its ability to release a 5′-dRP moiety. The K101A substitution almost completely abolished the 5′-dRP lyase activity (Fig. 6d), and the mutant was unable to form a covalent complex with the 5′-dRP-containing DNA in the presence of NaBH_4_ (see Fig. 6c). These results point to PgVPolX residue Lys101 as the main nucleophile responsible for the Schiff-base formation during the release of the 5′-dRP group.

**Figure 7:**
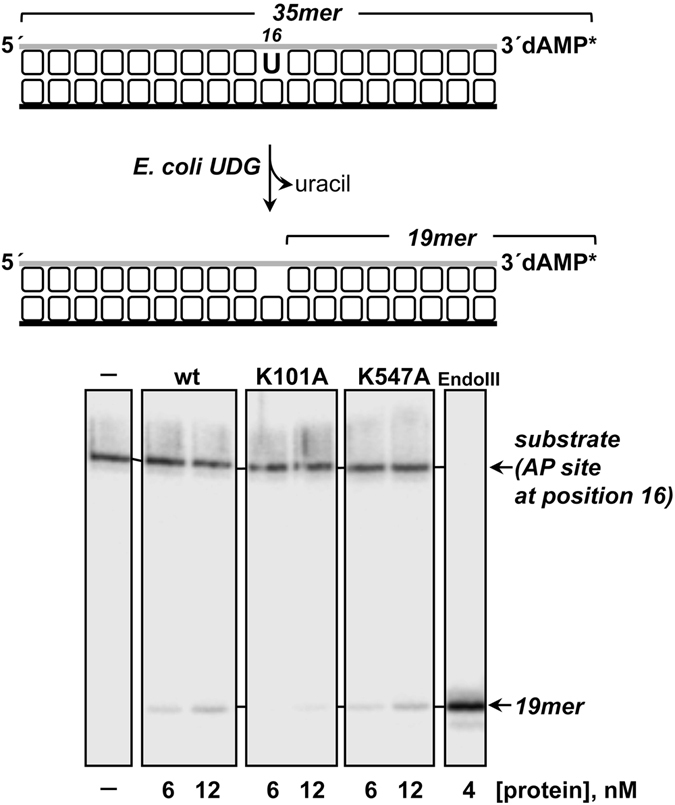
*AP-lyase activity of PgVPolX*. The depicted [^32^P]3′-labeled uracil-containing oligonucleotide (top) was treated with *E. coli* UDG, leaving an intact AP site. The resulting AP-containing DNA (1 nM) was incubated in the presence of either *E. coli* EndoIII that incises 3′ to the AP site, or the indicated PgV-PolX for 10 min at 30 °C, as described in Materials and Methods. Position of products is indicated.

The 5′-dRP lyases that follow a β-elimination mechanism have been defined as a subset of AP-lyases, and they could potentially be capable of cleaving unincised AP sites^52^. Thus, to study whether PgVPolX exhibits lyase activity on unincised AP substrates, we used a substrate consisting of a 34-mer dsDNA containing a 2′-deoxyuridine at position 16 of the 3′-labeled strand and treated this with UDG to form a natural AP site (see scheme in Fig. 7). The incubation of the AP site-containing DNA with increasing concentrations of either the wild-type or the ligase-deficient mutant K547A, in the absence of divalent cations, rendered a nicked product with an electrophoretical mobility identical to that produced by the AP-lyase activity of *E. coli* endonuclease III (EndoIII) that incises at the 3′-side of the AP site^3^. By contrast, the 5′-dRP lyase-deficient K101A mutant was severely impaired in its ability to incise on the internal AP site (Fig. 7). These results are consistent with cleavage of the phosphodiester bond at the 3′ side of the AP site in a metal-independent manner, leading us to infer the presence of an intrinsic AP-lyase activity in PgVPolX that relies on the same active site as does the 5′-dRP lyase.

### Repair of a single nucleotide gap

**Figure 8:**
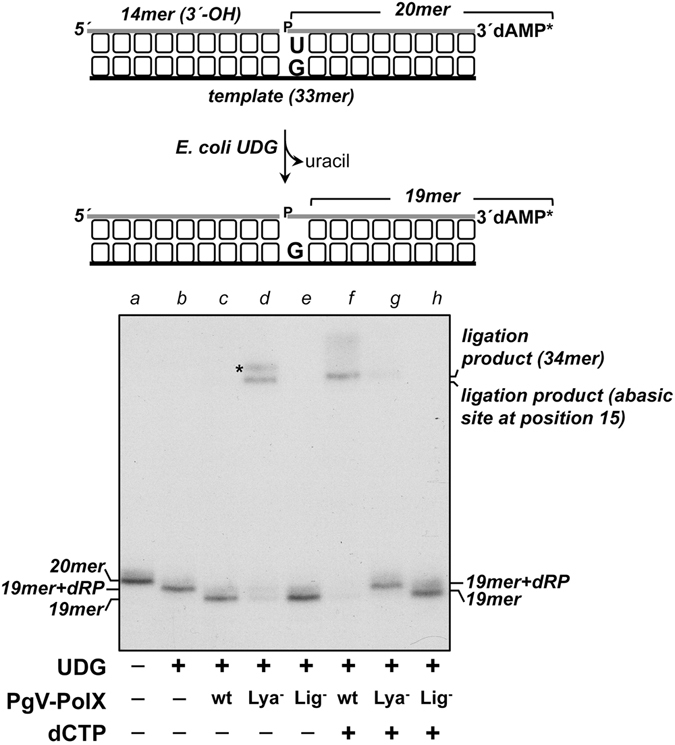
*In vitro* repair of a single nucleotide BER intermediate. Top, schematic representation of the formation of the BER intermediate used as substrate. The [^32^P]3′-labeled 2′-deoxyuridine-containing substrate (lane *a*) was treated with 27 nM *E. coli* UDG (lane *c*), leaving a 5′-dRP terminus. The resulting BER intermediate (1 nM) was incubated with 3 nM of the indicated PgVPolX in the absence (−) or presence (+) of 10 µM dCTP for 10 min at 30 °C, as described in Materials and Methods. Position of products is indicated. Asterisk indicates an electrophoretic artifact that appears specifically with the 34mer product with an AP site at position 15.

The efficient DNA polymerization activity exhibited by PgVPolX on gapped molecules, together with its intrinsic 5′-dRP lyase and ligase activities, strongly suggests that PgVPolX would be able to conduct the three last steps of the single nucleotide BER pathway. The above results led us to gauge the competence of the enzyme to complete the repair of a gapped BER intermediate, where the gap is flanked by a 3′-OH and a 5′-dRP group. A hybrid DNA containing a nick flanked by an upstream 3′-OH-ended 14mer strand (see scheme in Fig. 8) and a 20mer downstream strand with a 5′-phospho 2′-deoxyuridine (lane *a*) was treated with UDG to generate, opposite to dGMP in the template strand, a natural 5′-dRP-end that remained stable throughout the assay (lane *b*). The 5′-dRP lyase activity of both the wild-type and the ligase-deficient mutant K547A (Lig^−^ in Fig. 8) rendered a 19mer product after the release of the 5′-dRP moiety (lanes *c* and *e*). Importantly, the 5′-dRP lyase-deficient K101A mutant (Lya^−^ in Fig. 8) produced a ligation product (lane *d*), indicating that the ligase activity of PgVPolX can accomplish direct sealing of the 3′-OH and 5′-dRP groups. The absence of ligation products with the wild-type enzyme (lane *c*) would suggest that elimination of the 5′-dRP moiety by its lyase activity is faster than the ligation of both ends. Thus, PgVPolX converts the nicked molecule into a 1-nt gapped intermediate, preventing the regeneration of an internal AP site. As expected, in the presence of 10 µM dCTP, the wild-type enzyme produced a repaired 34mer product arising from the filling of the 1-nt gap and further sealing of the resulting nick (lane *f*). Under these conditions, the K101A mutant failed to produce any ligation product (lane *g*), which implies that the elongated upstream strand cannot be ligated to the dangling 5′-dRP group. Therefore, the three activities, polymerization, 5′-dRP lyase and DNA ligase act in concert to allow PgVPolX to efficiently accomplish the gap-filling, 5′-dRP excision and sealing steps during the repair reaction.

## Discussion

Due to the enormous diversity of genetic lesions, no single repair process can efficiently repair all types of DNA damage. Five known systems, BER, nucleotide excision repair, double-strand break repair, mismatch repair and direct damage reversal, which all arose early in evolution and are highly conserved across prokaryotes and eukaryotes^2^, are specialized pathways dedicated to repairing specific types of lesions. As their cellular homologs, viral genomes are also continuously exposed to exogenous genotoxicants and to by-products of host metabolism such as the ROS. These molecules can cause a plethora of lesions and lead to irreversible mutations, altered gene expression, and chromosomal aberrations, as well as to blockage of replication and transcription^2^. Interestingly, whereas the aforementioned repair functions are infrequent or absent in small viral genomes, enzymes belonging to the specialized pathways are present in the NCLDVs^33, 34^. PgV-16T is a giant virus belonging to the NCLDV group^35, 53^ and its genome contains the sequences for an almost complete BER pathway. Among the proteins encoded by the PgV-16T genome, one that merits attention is a bimodular protein, PgVPolX, with an N-terminal Polβ-like core and a C-terminal NAD^+^-dependent DNA ligase. The biochemical properties displayed by PgVPolX enable it to play an active role in BER as it shows a distributive polymerization pattern on template/primer molecules. Moreover, it is particularly active on gapped structures harboring a downstream 5′-P group whose recognition by the protein enables it to fill-in the gap processively and accurately. As shown for Pol β and λ, PgVPolX has an inherent 5′-dRP lyase activity dependent on residue Lys101 of the N-terminal 8-kDa domain, which is homologous to the nucleophiles Lys72 and Lys310 of Pol β and λ, respectively, and is responsible for the Schiff-base formation during elimination of the 5′-dRP group. The presence of both polymerization and 5′-dRP lyase activities in DNA polymerases, as in family X Pol β and λ, family A Pol γ and θ, and family Y Pol ι, is a compelling argument for a role for these enzymes in BER^18, 19, 49–51^. In addition, we have established the presence of an NAD^+^-dependent DNA ligase activity in PgVPolX that acts in concert with the polymerization and 5′-dRP lyase activities to accomplish the last three steps during “short” BER, most likely after the action of the viral AP-endonuclease. Alternatively, the presence of an intrinsic AP-lyase activity in PgVPolX suggests that this enzyme could act on unincised AP sites in the viral DNA, cleaving at the 3′-side of the AP site and consequently leaving a non-extendable 3′-phospho-α,β-unsaturated aldehyde (PUA) end. If this is the case, further filling of the gap would rely on previous processing of the 3′-blocked end by a phosphodiesterase activity. In this regard, the *P. globosa* virus genome contains an ORF coding for a hypothetical Nth-like AP-endonuclease, whose cellular counterparts are endowed with an additional phosphodiesterase activity that processes PUA-containing 3′-ends. Thus, the concerted action of the viral AP-endonuclease and PgVPolX would be sufficient to efficiently repair AP lesions by those alternative BER pathways.

Aside from the above-mentioned ORFs, the *P. globosa* virus genome has ORFs coding for putative sliding clamps and Flap endonucleases^34, 36^ whose cellular homologs play critical roles in the eukaryotic long-patch BER pathway^3^. This fact, together with the ability of PgVPolX to perform limited strand displacement suggests that AP sites could be repaired by a PgVPolX-mediated long-patch BER when the 5′-dRP group in the gap cannot be processed, as is proposed to occur with Pol β^54^.

Although an increasing number of giant viruses have a gene coding for a putative PolX^34^, the amino acid sequence alignment of these viral PolXs show that the nucleophilic lysine responsible for the 5′-dRP lyase is not conserved (Supplementary Fig. S2). Interestingly, in many of these cases, the viral genome contains ORFs coding for both bifunctional *N*-glycosylases, which could cleave at the 3′-side of the AP site, and AP-endonucleases^34^. The sequential action of these proteins would render a 1-nt gapped intermediate further filled-in by the polymerase and the resulting nick sealed by the ligase.

Sequencing of the pangenome of giant DNA viruses has revealed the presence of genes encoding for most of the critical enzymes involved in DNA repair processes^33, 34^. Recent evolutionary studies have revealed that those genomes show a nonsynonymous/synonymous substitution rate below one, suggesting that most of the genes in these types of viruses are functional and contribute to virus adaptation^33, 55^. These observations could emphasize the importance of the preservation of those viral DNA repair functions to slow down viral evolution by safeguarding their genomes from random alterations^55^. In summary, we have demonstrated the functionality of the first PolX-NAD^+^-dependent DNA ligase fusion reported, and how its activities are compatible with DNA repair pathways. Our findings support the notion that other viral protein fusions, such as the fused histones in *Lausannevirus* and *Marseillevirus* ^56^ or the AP-endonuclease-PolX fusion in *Entomopoxviruses* ^57^, will also be active.

## Materials and Methods

### Proteins, reagents and oligonucleotides

Unlabeled nucleotides were purchased from GE Healthcare. [α^32^P]cordycepin (3′-dATP) and [γ^32^P]ATP were obtained from Perkin Elmer Life Sciences. Where indicated, substrates were radiolabeled at the 3′ end with [α^32^P]cordycepin using TdT or, at the 5′ end, with [γ^32^P]ATP using T4 polynucleotide kinase (T4PNK). TdT, T4PNK, *E. coli* uracil DNA glycosylase (UDG) and *E. coli* EndoIII were from New England Biolabs.

Recombinant PgVPolX: The ORF corresponding to gene 401 of *P. globosa* virus was synthesized by Genscript Corporation and cloned between the *Nde*I and *Bam*HI restriction sites in the pET16b vector to express a recombinant protein fused to an N-terminal (His)_10-_tag for purification on Ni^+2^-affinity resins. *E. coli* BL21 (DE3) cells, which contain the T7 RNA polymerase gene under the control of the isopropyl β-D-thiogalactopyranoside (IPTG)-inducible lacUV5 promoter^58, 59^, were transformed with the resulting recombinant expression plasmid, named pET16-PgVPolX. Transformed cells were grown overnight at 37 °C in LB medium with ampicillin (100 µg/ml). Cells were diluted into the same medium and incubated at 37 °C until the OD_600_ reached 0.6. Then, IPTG (Sigma) was added to a concentration of 0.5 mM and incubation was continued for 16 h at 15 °C. Cells were collected by centrifugation for 10 min at 6143 × *g*. Cells were thawed and ground with alumina at 4 °C; the slurry was resuspended in Buffer A (50 mM Tris-HCl, pH7.5, 1 M NaCl, 7 mM β-mercaptoethanol, 5% glycerol) and centrifuged for 5 min at 650 × *g* at 4 °C to remove alumina and intact cells. Recombinant PgVPolX protein was soluble under these conditions since it remained in the supernatant after a further centrifugation step for 20 min at 23,430 × *g* to separate insoluble proteins from the soluble extract. The DNA present was removed by stirring the soluble extract containing 0.3% polyethyleneimine for 10 min followed by centrifugation for 10 min at 23,430 × *g*. The resulting supernatant was precipitated with ammonium sulphate to 70% saturation, to obtain a polyethyleneimine-free protein pellet. After centrifugation for 25 min at 23,430 × *g*, the pellet was resuspended in Buffer A without NaCl to give a final ammonium sulphate concentration of 300 mM. This fraction was loaded onto a phosphocellulose column pre-equilibrated with Buffer A (300 mM NaCl) and the PgVPolX protein was step-eluted with 300, 400, 425, 450, 475, 500 and 600 mM NaCl. The His-tagged PgVPolX was recovered in the 475 and 500 mM NaCl eluate fractions, which were then loaded onto a Ni-NTA column (Qiagen) pre-equilibrated with Buffer A (500 mM NaCl and 5 mM imidazole). The affinity column was step-eluted with 20, 50, 75, 100, 125, 150, 175 and 200 mM imidazole in Buffer A (300 mM NaCl). The polypeptide composition of the column fractions was monitored by SDS-PAGE. The His-tagged PgVPolX was recovered in the 100 and 125 mM imidazole eluate fractions. Finally, PgVPolX was dialyzed against a buffer containing 50 mM Tris-HCl, pH 7.5, 1 mM EDTA, 7 mM β-mercaptoethanol and 50% glycerol and stored at −20 °C. The final purity of the protein was estimated to be >90% by Coomassie blue-stained SDS-PAGE. The purified protein was further loaded onto a 4-ml glycerol gradient (15–30%) containing 50 mM Tris-HCl, pH 7.5, 20 mM ammonium sulphate, 180 mM NaCl, 1 mM EDTA, and 7 mM β-mercaptoethanol, and centrifuged at 4 °C for 24 h at 58,000 rpm in a Beckman TST 60.4 rotor. After centrifugation, 34 fractions were collected from the bottom of the tube for further analysis.

Recombinant PgVPolX polymerization domain: Plasmid pET16-PgVPolX (see above) was used as a template to generate the N-terminal polymerization domain (residues 1–381) by changing codon 382 (GAA) into the stop codon TAA using the QuikChange^®^ site-directed mutagenesis kit (Stratagene). Confirmation of the DNA sequence and the absence of additional mutations was carried out by sequencing the entire gene. BL21 (DE3) cells were transformed with the resulting plasmid and induction of protein expression and preparation of soluble bacterial lysates were performed as described for full-length PgVPolX. The supernatant containing the PgVPolX polymerization domain was loaded onto a Ni-NTA column pre-equilibrated with Buffer A (300 mM NaCl and 5 mM imidazole). The affinity column was step-eluted with 50, 100, 150, 175 and 200 mM imidazole. The polypeptide composition of the column fractions was monitored by SDS-PAGE. The His-tagged PgVPolX-polymerization domain was recovered in the 100 mM imidazole-eluate fraction and was loaded onto a phosphocellulose column pre-equilibrated with Buffer A (300 mM NaCl). The His-tagged PgVPolX-polymerization domain was step-eluted with Buffer A (50, 500 and 600 mM NaCl). The protein was recovered in the 600 mM NaCl eluate fraction and further dialyzed against a buffer containing 50 mM Tris-HCl, pH 7.5, 1 mM EDTA, 7 mM β-mercaptoethanol, 0,025% Tween^®^-20 and 50% glycerol and stored at −20 °C.

PgVPolX catalytic-deficient mutants*:* PgVPolX mutants D245A/D247A, K101A and K547A were obtained using the QuikChange^®^ system. Plasmid pET16-PgVPolX was used as a template for mutagenesis. PgVPolX mutants were purified from soluble extracts of IPTG-induced BL21(DE3) cells by phosphocellulose and Ni-NTA chromatography as described for the wild-type enzyme.

Oligonucleotides were purchased from Sigma-Aldrich (sequences are listed in Table 1). When indicated, oligonucleotides were radiolabeled either at the 5′ end using [γ^32^P]ATP (3000 Ci/mmol) and T4PNK or at the 3′-end using [α^32^P]cordycepin and TdT. Substrates were annealed in the presence of 60 mM Tris-HCl (pH 7.5) and 0.2 M NaCl at 80 °C for 5 min before slowly cooling to room temperature overnight.

**Table 1:** Oligonucleotides used in this study.

Name	Sequence (5′-3′)
P	GATCACAGTGAGTAC
Cy5P	(Cy5)GATCACAGTGAGTAC
DOH	AACGACGGCCAGT
DowP	(p)AACGACGGCCAGT
T33	ACTGGCCGTGCTTCTATTGTACTCACGTGATCT
T29	ACTGGCCGTGCTT**X**GTACTCACTGTGATC
T28	ACTGGCCGTGCTTGTACTCACTGTGATC
pU	CTGCAGCTGATGCGC**U**GTACGGATCCCCGGGTAC
pUc-G	GTACCCGGGGATCCGTACGGCGCATCAGCTGCAG
PC	GTACTCACTGTGATC
PddC	GTACTCACTGTGAT(ddC)
PC-1	GTACTCACTGTGAT
Ph	(p)**U**AGCTGATGCGCAGTACGG
DU	CCGTACTGCGCATCAGCTGATCACAGTGAGTAC

U denotes 2′-deoxyuridine.

Cy5 stands for 1,1′-bis(3-hydroxypropyl)-3,3,3′,3′-tetramethylindodicarbocyanine dye.

X = A, C, G or T.

(p) = 5′-phosphate group.

### DNA polymerization assays on defined DNA molecules

DNA-dependent polymerization was assayed on a template/primer substrate (obtained by hybridization of oligonucleotides Cy5P and T33, see Table 1), on 5-nt gapped molecules (obtained by hybridization of oligonucleotides Cy5P, T33 and either DOH or DowP, which contains a 5′-phosphate) and on 1-nt gapped molecules (obtained by hybridization of oligonucleotides Cy5P, T29X and DowP). The incubation mixture (12.5 μl) contained 50 mM Tris-HCl pH 7.5, 1.25 mM MgCl_2_, 1 mM DTT, 4% (v/v) glycerol, 0.1 mg/ml BSA, 25 nM of the hybrid shown in each case, and the indicated concentrations of PgVPolX (or 2 µl of each of the PgVPolX containing fractions from the glycerol gradient) and nucleotides. After incubation for 5 min at 30 °C the reactions were stopped by adding EDTA to 10 mM. Samples were analyzed by 7 M urea-20% PAGE and visualized using a Typhoon 9410 scanner (GE Healthcare).

### Ligation of nicked DNA

To obtain a nicked DNA substrate, [^32^P]5′-labeled oligonucleotide P and DowP were hybridized to oligonucleotide T28 (see Table 1). The incubation mixture (12.5 μl) contained 50 mM Tris-HCl pH 7.5, 1.25 mM MgCl_2_, 1 mM DTT, 4% (v/v) glycerol, 0.1 mg/ml BSA, 1 nM of the nicked DNA substrate, and the indicated concentrations of both PgVPolX and NADH. After incubation for 5 min at 30 °C the reactions were stopped by adding EDTA to 10 mM. Samples were analyzed by 7 M urea-20% PAGE and autoradiography.

### 5′-dRP lyase activity on gap-filled BER intermediates

To obtain a gap-filled BER intermediate, the downstream [^32^P]3′-labeled oligonucleotide Ph, which contains a 5-phospho 2′-deoxyuridine, and either PC (upstream primer with a 3′-dCMP) or PddC (upstream primer with a 3′-ddCMP) were hybridized to oligonucleotide DU (see Table 1). A concentration of 1 nM of the hybrid molecules was further treated with 27 nM UDG for 15 min at 37 °C in the presence of 30 mM Hepes pH 7.5, 4% (v/v) glycerol, to render a 5′-dRP end. After incubation, the mixture was supplemented with the indicated concentration of PgVPolX, in the absence or presence of 1.25 mM MgCl_2_. Samples were incubated at 30 °C for the indicated times. After incubation, freshly prepared NaBH4 was added to a final concentration of 100 mM and the reactions were incubated for an additional 20 min on ice. Stabilized (reduced) DNA products were ethanol-precipitated with 0.2 µg/ml tRNA, resuspended in water and analyzed by 7 M urea-20% PAGE and autoradiography.

### NaBH_4_ trapping assay

A 6 nM concentration of the gap-filled BER intermediate with the upstream strand harboring a 3′-ddCMP end (see above) was treated with 27 nM UDG for 15 min at 37 °C in a mixture containing 30 mM Hepes pH 7.5, 4% (v/v) glycerol and 1.25 mM MgCl_2_. After incubation, the mixture was supplemented with 12 nM of the indicated PgVPolX protein and incubation continued for 15 minutes at 30 °C. The Schiff base intermediate was trapped by the addition of 100 mM NaCl or freshly prepared NaBH4. After incubation on ice for 20 min, samples were analyzed by 10% SDS-PAGE followed by autoradiography.

### AP lyase activity assay on 2′-deoxyuridine-containing substrates

To prepare dsDNA substrates with an internal AP site, [^32^P]3′-labeled oligonucleotide pU, which contains a 2′-deoxyuridine at position 16, was hybridized to the complementary oligonucleotide pUc-G (see Table 1). A 1 nM concentration of the hybrid was treated with 27 nM UDG for 15 min at 37 °C in the presence of 30 mM Hepes pH 7.5, 4% (v/v) glycerol. After incubation, the mixture was supplemented with either 4 nM of *E. coli* EndoIII, or the indicated concentrations of PgVPolX. Samples were incubated at 30 °C for 10 minutes and reactions were processed as described for the 5′-dRP lyase activity assay.

### *In vitro* reconstitution of single-nucleotide BER

To obtain the BER intermediate, the upstream oligonucleotide PC-1 and the [^32^P]3′-labeled downstream oligonucleotide Ph were hybridized to oligonucleotide DU (see Table 1). A 1 nM concentration of the hybrid molecule was incubated with 27 nM UDG in the presence of 30 mM Hepes pH 7.5, 4% (v/v) glycerol, 1.25 mM MnCl_2_, 50 mM NADH and, when indicated, 10 µM dCTP. After incubation for 15 min at 37 °C, 3 nM of the indicated PgVPolX was added. Samples were incubated at 30 °C for an additional 10 min; after which, freshly prepared NaBH_4_ was added to a final concentration of 100 mM, and the reactions were incubated for an additional 20 min on ice. Stabilized (reduced) DNA products were ethanol-precipitated with 0.2 µg/ml tRNA, resuspended in water and analyzed by 7 M urea-20% PAGE and visualized in a Typhoon 9410 scanner in phosphorimager mode.

## Electronic supplementary material

Supplementary Information
