# Supplementary material for: Phaeocystis globosa Virus DNA Polymerase X: a “Swiss Army knife”, Multifunctional DNA polymerase-lyase-ligase for Base Excision Repair
Source: Sci Rep. 2017 Jul 31;7:6907. doi: 10.1038/s41598-017-07378-3 (PMC5537341; doi:10.1038/s41598-017-07378-3)
Supplement: Supplementary file 1 — Supplementary Information [file 41598_2017_7378_MOESM1_ESM.pdf]

Supplementary Information

Phaeocystis globosa Virus DNA Polymerase X: a "Swiss Army knife", Multifunctional DNA polymerase-lyase-ligase for Base Excision Repair

José L. Fernández-García[1], Ana de Ory[1], Corina P.D. Brussaard[2] and Miguel de Vega[1,*]

[1]Centro de Biología Molecular Severo Ochoa (CSIC-UAM), C/Nicolás Cabrera 1, Campus Cantoblanco, 28049 Madrid, Spain.

[2]Department of Marine Microbiology and Biogeochemistry, NIOZ Royal Netherlands Institute for Sea Research, Utrecht University, NL-1790 AB Den Burg (Texel), The Netherlands

[*]To whom correspondence should be addressed: Miguel de Vega, Centro de Biología Molecular Severo Ochoa (CSIC-UAM), C/Nicolás Cabrera 1, Campus Cantoblanco, 28049 Madrid, Spain, Telephone: (+34) 911964717, Fax: (+34) 911964420, E-mail: mdevega@cbm.csic.es.

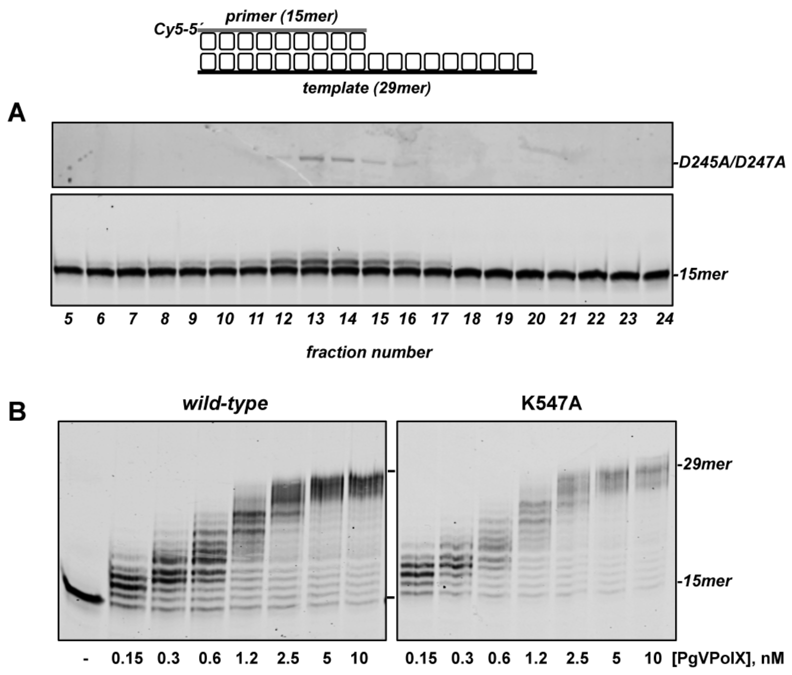

Supplementary Figure S1. (A) *Sedimentation analysis of the nucleotide insertion capacity of PgVPolX mutant D245A/D247A.* The upper panel shows the SDS-PAGE analysis followed by Coomasie Blue staining of the glycerol gradient fractions 5-24 of mutant D245A/D247A. The bottom panel shows the polymerization activity of the individual fractions. The assay was carried out as described in Materials and Methods using 25 nM of the primer/template substrate depicted on top of the figure, 2 µl of each fraction and 100 µM dNTPs. After incubation for 5 min at 30°C, the reactions were stopped by adding EDTA up to 10 mM. Samples were analyzed by 7 M urea-20% PAGE and visualized using a Typhoon 9410 scanner. The position of the primer is indicated. **(B)** *Polymerization activity of wild-type and mutant K547A PgVPolX on a template/primer substrate*. The assay was performed as described in Materials and Methods, in the presence of 25 nM of the primer/template substrate depicted on top of the figure and 100 µM dNTPs. After incubation for 5 min at 30°C, the reactions were stopped by adding EDTA up to 10 mM. Samples were analyzed as described in (A).

```
ASFV     MLTLI-Q------GK--KIVNH-------------------------------------------------------------------------------
PgV16T   MRSIK-Q---------NKRIKKKSQTKKQKLKGLKVKSHIKNMNGGMKDYKNEYIEILKQLEYYNRVKEKAPFKAKIYREAV--------EQIKNLNGE--
CeV      MSKTLKQQLIIKTNSSNKLKLSNSAEKLKKKSLTPENIEAKSMSQSKRYNEEFIEVLGELAQL--MIKKGEPFRSRAYQKA--------EEAIIKFPDD--
APMV     MNSKI-I------EQFNLLEKQVDA---------------------------EYLN--SKVENDLKEETMNRFRLKSIKKAL--------SILKNLDFE--
Mmon     MNANI-I------EQFSLLVKQIEA---------------------------EYLN--AQVENDVKEINNHKFRLKSVKEIL--------GILKRLDFE--
Mchi     MNSNI-I------EQFILLVKQIEA---------------------------EYLN--AQVENNPKEISAHKFRLQSNKRIL--------GIIKNIDFE--
CroV     MNSNV-I------SQFEKLVSKNQQ---------------------------DLDN--AIKNKLTDEQRKQSFKLRTNKRVL--------SIIKKYPEEL-
MSenPv   NISNIDL------NTFMAILNKRKI---------------------------IYML--HNIANIYEKLDENKYK-AYNKAINSIYEQGIIILKYNNTTIN-
AMenPv   MKIYLDIKQNMNIDNFMSMINKHKI---------------------------LLVL--NKFADIYNILNEIKYK--AFLNAAY--------VIQNTPVIIF
Pol β    MSKRK-A------PQETLNGG---I---------------------------TDML--TELANFEKNVSQAIHKYNAYRKAA--------SVIAKYPHK--

ASFV     ------------------------------------------------LR---------------------------------------------------
PgV16T   ----------------VNSADDIKGLSGIGKAITDKLTEFINTGQVKNLEKLKSEYNTEDYENEKIKQAKKDIFLQIHGIGDAAAEKLID-LGITTIEELK
CeV      ----------------IYNIKQLKNIPGIGTTILTKLEEYIKTGKVSVLERERND--------------PANILAGIYGIGPKKAKELVE-SGIISIAQL-
APMV     ----------------ITDANDVKGIPGIGAGTIKRIKEILETGKLHDLKDK---FSPEK--QKQIEG--IQELENVIGIGSSTAKKLISQYGIRSVDDLK
Mmon     ----------------ITDPSDLDGIRGIGTSTKRRIAEILETGKLSELKNK---YDKKN--NRKM-----------------------------------
Mchi     ----------------ITDPSDLNGIQGIGDGTKRRIAEILETGKLSELKNK---YDKKK--QEKINS--IQELERVIGIGSSTAKKLITKYGITSVKELK
CroV     ---------------TINNYKELGDLNGIGKGTITKLQNIFEKGYIDELKGY---TAPK---NNKEDI--IKNLEEVINIGRSTAKELVE-SGVKSVDDLK
MSEnPv   SPKLVD--------NMETVISKYKDIPNIGDSISKKIYNIISNTYIDNISNT-----------KEYKY--ITELMNVLYIGPKTAKNLIS-KGIKTIKDLK
AMenPv   KYKNVNNKFILN-ESKENIIQKYKNLKSIGTSISDIIYELLSTNKVEKLINL---EN-----NSSYKY--IKILTSILFIGPKKAQSLLK-LNIKNINDLI
Pol β    ----------------IKSGAEAKKLPGVGTKIAEKIDEFLATGKLRKLEKI---RQ-----DDTSSS--INFLTRVSGIGPSAARKFVD-EGIKTLEDLR

ASFV     ---------------------------SRLAFEYNGQLIK--------------------------IL--SK-NIVAVGSLRREEKMLNDVDLLIIVPEKK
PgV16T   ARKNEEIPGKGTKKLKLLNAT------QQKGLEYYEEILEKIPRAEIEEYKTTIEKLFN--EATDNNTED--NKFEIVGSYRRGKQESGDIDIIITSKVND
CeV      ----HDEPG-----IDMLNEK------QKLGVKYYEDIEKKIPRAEIVEFRELFKQLFD--KVAPKNSD-----FEIVGSFRRGAKTSGDIDIIITNKNNN
APMV     KAIET-------GKVK-VSTS------IMLGLKYYGIVQRDIPRKEITAIEKLLSK------EAHKIDPD--LEIIICGSYRRGKKTSGDIDVLMYHPKMK
Mmon     -----------------------------------------------------------------------------------------------YHRAMK
Mchi     KAIKS-------GEVE-VSNA------ILLGLKYYGIVEGNIPRKEIDQVEKYLEK------QAEKINED--LEIMICGSYRRGKATSGDIDVLIYHPGMK
CroV     KKVNT-------KKIE-VNSK------ILLGLKYYQKIQERIPRNHITEINEFIKNRINFLNKKEKLTAENKYIFKICGSYRRNKPTSGDIDILITQKNTS
MSEnPv   KNKD-----------KYLNSQ------QQIAIEYYDKL-KPINRSFISNLES-----------SIKLSPN--KEWYILGSYARGKLTSKDIDILIVDFDID
AMenPv   EKKD-----------NIINMGILTIH-EIKIIEYIKDM-EPVSRNFINDLKQ-----------NINLSSE--CEWYILGSYARGLDYSKDIDILIIDFTID
Pol β    KN-----------EDK-LNHH------QRIGLKYFGDFEKRIPREEMLQMQDIVLN------EVKKVDSE--YIATVCGSFRRGAESSGDMDVLLTHPSFT

ASFV     LLKHVL----------------PNIRIKGLSFSVKVC-GERKCVLFIEWE------K-KTYQLDLFTALAEEKPYAIFHFTGPVSYLIRIRAALKKKNYKL
PgV16T   KT--------------SFDKFLDLLKEKDIIKVFLSR-GEKKSMVISKLNS-----ESTARRLDFLYTPPEEYAFAILYFTGSKDFNTGMRMLALKNNLTL
CeV      RE--------------AFDKVLDLLIRDKIITEVLSRGKTKSLTLVQIKKD------APIRRVDFLYTPPDEYAFALFYFTGSKLFNTIVRQKALDLGYTL
APMV     TSKEM-LHPEKFDLEPYFNLYIDRLTEKGFLIDDITFNPNKKYMGFCKYK------LNPVRRIDIRFIPYNSLAPAMLYFTGPMELNTKMRSAAKKRKMIL
Mmon     TTKEI-LNHEKYNLEPYFEMYIGQLTNNGFLLDHMTDAATKKYMGFCKYK------SYPVRRIDIRFVPYQSLPSAMLYFTGPYELNTIMRSAAKKRKMIL
Mchi     TTKEM-LNPEKYDLEPYFEIFISNLTKNGFLLDHMTDGSNKKYMGFCKYK------SNPVRRIDVRFIPYQSLPSAMLYFTGPYELNTIMRSSAKKRKMIL
CroV     KTSKY-LDEHLPKIIQMFKQPWKGNNFKPFLIDNLTDITPTKYMGFAQFK------NNLPFRIDIRFVSYDSFYTALLYFTGSDEFNKVMRNIAKDKGYKL
MSEnPv   E-------------------ILKEIYKYAKEEAVLRH-GKIMYSGIFKKD-------KTYFLIDIYKTTESEKYTSIQYFTGSKEFNIALRSLALSKDIRL
AMenPv   K-------------------FLEELKKIAKLMYIIRK-GNNIFSGVFLWQ-------GKKFILEINKVNNKEKYTAIMHFTGSKKFNIFMRNIAKSENMIL
Pol β    SEST--------KQPKLLHQVVEQLQKVHFITDTLSKG-ETKFMGVCQLPSKNDEKEYPHRRIDIRLIPKDQYYCGVLYFTGSDIFNKNMRAHALEKGFTI

ASFV     NQYGLFKNQT------LVPLKITTEKELIKELGFTYRIPKKR
PgV16T   NEHGFHTMKDKIKGDKITSPEFNTEEDIFKYLNMEFKEPHERL
CeV      NEHGLSYMSKGIKGKKV-DESFPTEASILNFLGFEYVKPENRVD
APMV     NEYGLFKTDKN--GA-QIPLDTKSEADIFHALGMDYLTPQQRELYSSGKIH
Mmon     NEYGLYKVDEN--GL-KIPVKIKSEADIFNALGMDYLTPEERESYNTGKVSKAK
Mchi     NEYGLYKVDAN--EI-KTPVKIKSEADIFNALGMDYLTPQERETFNTGKINKSK
CroV     SEYGLFKIDS------KNQVPIKSEEHVFKILNMDYLEPHLR
MSEnPv   NQHEMYDLQT------QKKYNIKSEKDIFDILGVKYVPPSKRDIYTTV
AMenPv   NQYSLKKDNV--------ELPITKEEDIFDYLKIKYIPNNKRNI
Pol β    NEYTIRPLGVT--GVAGEPLPVDSEKDIFDYIQWKYREPKDRSE
```

Supplementary Figure S2. *Multiple amino acid sequence alignment of the Pol β-like core of representatives of NCDLV with human Pol β.* Names of virus are abbreviated as follows (numbers in parentheses indicate the corresponding accession number): ASFV, African Swine Fever Virus (NP_042790.1); APMV, *Acanthamoeba polyphaga* Mimivirus (YP_003986821.1); Mmon, Moumovirus monve (AEX62672.1, AEX62673.1); Mchi, *Megavirus chiliensis* (YP_004894637.1); CroV, *Cafeteria roenbergensis* virus (YP_003970091.1); MSenPv, *Melanoplus sanguinipes* entomopoxvirus (residues 275-603; NP_048188.1); AMenPv, *Amsacta morei* entomopoxvirus (residues 277-612; NP_064992.1). White letters boxed in black indicate the three conserved Asp responsible for the polymerization reaction. Yellow letters boxed in black indicate the proposed nucleophile residue responsible for the 5´-dRP lyase activity. The alignment is divided in four subdomains: 8-kDa (green area), fingers (yellow area), palm (pink area) and thumb (blue area).
